# To Detach, Migrate, Adhere, and Metastasize: CD97/*ADGRE5* in Cancer

**DOI:** 10.3390/cells11091538

**Published:** 2022-05-04

**Authors:** Gabriela Aust, Leyu Zheng, Marianne Quaas

**Affiliations:** 1 Research Laboratories of the Clinic of Visceral, Transplantation, Thoracic, and Vascular Surgery, Medical School, University Hospital Leipzig, Leipzig University, 04103 Leipzig, Germany; gabriela.aust@medizin.uni-leipzig.de; 2 Research Laboratories of the Clinic of Orthopedics, Traumatology and Plastic Surgery, Medical School, University Hospital Leipzig, Leipzig University, 04103 Leipzig, Germany; leyu.zheng@medizin.uni-leipzig.de

**Keywords:** CD97, ADGRE5, EGF-TM7, aGPCR, cancer, tumor, migration, invasion, metastasis, angiogenesis

## Abstract

Tumorigenesis is a multistep process, during which cells acquire a series of mutations that lead to unrestrained cell growth and proliferation, inhibition of cell differentiation, and evasion of cell death. Growing tumors stimulate angiogenesis, providing them with nutrients and oxygen. Ultimately, tumor cells invade the surrounding tissue and metastasize; a process responsible for about 90% of cancer-related deaths. Adhesion G protein-coupled receptors (aGPCRs) modulate the cellular processes closely related to tumor cell biology, such as adhesion and detachment, migration, polarity, and guidance. Soon after first being described, individual human aGPCRs were found to be involved in tumorigenesis. Twenty-five years ago, CD97/*ADGRE5* was discovered to be induced in one of the most severe tumors, dedifferentiated anaplastic thyroid carcinoma. After decades of research, the time has come to review our knowledge of the presence and function of CD97 in cancer. In summary, CD97 is obviously induced or altered in many tumor entities; this has been shown consistently in nearly one hundred published studies. However, its high expression at circulating and tumor-infiltrating immune cells renders the systemic targeting of CD97 in tumors difficult.

## 1. Introduction: CD97—A Retrospect

GPCRs are seven-pass transmembrane (7TM) receptors that transduce signals derived from very distinct ligands outside of cells to intracellularly-associated heterotrimeric G proteins. Among the hundreds of different GPCRs, which are biosensors participating in multifaceted bodily functions, the 33 aGPCRs in humans are exceptional in structure, signaling modes, and functions. The unique structural features of aGPCRs include their unusually large extracellular domain (ECD), which contain many consecutive adhesive folds that facilitate adhesion (thus, naming these receptors), the presence of a tethered agonist (TA) hidden within a GPCR autoproteolysis-inducing (GAIN) domain, and their non-covalent heteromeric two-subunit layout.

The very first aGPCR discovered was mouse F4/80, which was cloned in 1981 [1]. Its orthologue in humans was later named EMR1. In 1994, CD97 became the first human aGPCR described, and cloned soon afterwards [2,3]. Strongly expressed on leukocytes, the receptor was included into the cluster of differentiation (CD) categorization of immune cell surface antigens and given the number 97 [4].

Based on (genome) structure, CD97/*ADGRE5*, EMR1/*ADGRE1* [5], EMR2/*ADGRE2* [6], and EMR3/*ADGRE3* [7] were classified next as EGF-TM7 receptors [8,9]. Their adhesive folds are tandemly arranged epidermal growth factor (EGF)-like repeats, compromising 30–40 amino acids found in many types of proteins that mediate cell-adhesive interactions. When the (phylogenetic) sequence relations and topology became clear after the sequencing of the human genome, aGPCRs/class B2 GPCRs were identified as a unique GPCR class [10,11,12]. The former EGF-TM7 receptors comprised their own subfamily and obtained an “E”, taken from EGF, for the new gene name *ADGRE* [13]. Here, we use the familiar “CD97” to designate the protein, and *ADGRE5* for the gene that encodes it. This review first provides a brief overview on the structure, interaction, and expression of CD97, and then assesses the established links between CD97 and cancer.

## 2. Overview of CD97/*ADGRE5*

### 2.1. CD97-A Prototypic aGPCR by Structure

**Figure 1:**
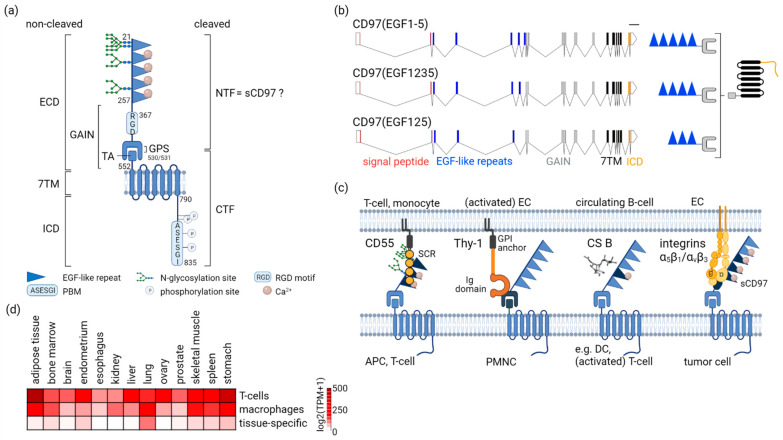
**Common features of human CD97**. (**a**) **Schematic structural organization of CD97.** The figure depicts the 835 amino acid full-length CD97(EGF1-5) isoform without the signal peptide. CD97 has a tripartite structure with the ECD, containing tandemly-arranged EGF-like repeats and the GAIN domain, the 7TM helices, and the ICD. The potential N-glycosylation sites in the EGF-like repeats are indicated. EGF2-5 can bind Ca^2+^, which is important for CD97 interactions. CD97 is self-cleaved at the GAIN domain-covered GPS, resulting in a bipartite structure with the NTF and CTF. Circulating sCD97 likely is the released NTF. The N-terminus of the CTF represents the integrated TA sequence. The ICD contains many phosphorylation sites. Four are confirmed in more than five records in which this modification was assigned using proteomic discovery mass spectrometry (phosphosite.org, accessed on 4 February 2022). In CD97(EGF1-5), these sites are S818, T825, S831, and S833. The ICD ends up in a PBM. For abbreviations and further explanations, see the running text. (**b**) **Exon/intron and protein structure of the three CD97/*ADGRE5* isoforms.** Alternative splicing results in isoforms with three to five EGF-like repeats with distinct binding properties. The isoforms are named CD97(EGF125), EGF(1235), and EGF(1-5), according to the EGF-like repeats present (wormweb.org, accessed on 15 March 2022). Intron: line; exon: solid bar; 5′/3′-UTR: empty bar; signal peptide: red; EGF-like repeats: blue; GAIN: grey; 7TM: black; ICD: orange; scale bar: 1000 base pairs (**c**) **Interaction partners with the CD97 ECD.** CD55 is a glycophosphatidylinositol (GPI)-anchored transmembrane receptor with four short consensus repeats (SCRs). The first three SCRs interact with at least three EGF-like repeats of CD97. Thy-1/CD90 is a small, heavily N-glycosylated GPI-anchored transmembrane receptor with a single extracellular immunoglobulin (Ig) domain. Thy-1 binding to CD97 on polymorphonuclear cells (PMNC) is calcium-independent and occurs through the GAIN domain. Binding to the glycosaminoglycan side chain chondroitin sulfate B (CS B), a component of the extracellular matrix and of cell surfaces proteoglycans, is mediated by the fourth EGF-like repeat of CD97. Thus, CS B interacts only with CD97(EGF1-5). Binding is Ca^2+^-dependent. Soluble recombinant CD97 interacts with integrins on endothelial cells (ECs) via its RGD motif and at least three EGF-like repeats. CS B can act synergistically. Interacting domains are indicated in dark blue. (**d**) ***ADGRE5* scRNAseq analysis in healthy normal human tissues.** The analysis comprises all protein-coding genes in 144 individual cell type clusters (The Human Protein Atlas, proteinatlas.org; accessed on 7 February 2022). In the heat map, only tissues containing the clusters “T-cells” and “macrophages” are considered. Additionally, the tissue-specific cell type cluster with the highest *ADGRE5* level is included (e.g., fat/adipocytes, lung/alveolar cells type 2, endometrium/smooth muscle cells, spleen/plasma cells, stomach/gastric mucus-secreting cells). Log2 transcripts per million (TPM) + 1 values are given. The figure was created with BioRender.com (accessed on 26 April 2022).

Hereinafter, the structural elements of CD97, starting with the extracellular N-terminus, and their functional relevance are described (Figure 1a).

Sixteen *ADGRE5* splice variants, of which six are protein-coding transcripts, are annotated (ensembl.org; accessed on 04 February 2022). Three out of six (3/6) coding transcripts are identical between the automatic Ensembl- and the manual Havana annotation. They encode alternative-splice variants within the EGF-like repeat region, resulting in three CD97 isoforms, which differ in their number of these repeats [14] (Figure 1b).

CD97 variability in the EGF-like repeats is enhanced through cell type-specific glycosylation, which regulates cellular adhesion. CD97 only binds CD55, one of its interaction partners, at the ECD, if the two *N*-glycosylation sites in the first, and one site in the, second EGF-like repeat are occupied [15]. Therefore, CD97 is N-glycosylated in leukocytes and many tumors, but not in smooth and skeletal myocytes [16,17].

One-hundred and ten amino acids at the end of the last EGF-like repeat follow an Arg-Gly-Asp (RGD) tripeptide sequence (Figure 1a) in humans (though not in mice), a common pattern in numerous proteins supporting cell adhesion, especially to the extracellular matrix (ECM). This motif is involved in CD97 binding to integrins on endothelial cells during tumor angiogenesis [18,19] and CD97-promoted adhesion, and the viability of fibrosarcoma HT1080 cells [20].

An exceptional structural feature of the aGPCR’s ECD is the GAIN domain covering the GPCR proteolytic site (GPS) [21] (Figure 1a). Autocatalytic cleavage at the GPS results in non-covalently bound N-terminal (NTF) and C-terminal fragments (CTF), formerly described as α- and β-chain for CD97 [14]. Endogenous and ectopic CD97 are normally cleaved completely.

The experimental observation that deletion of the NTF (ΔNTF) increases the basal activity of CD97 and that of other aGPCRs [22,23,24] is crucial for understanding their activation. Deleting the NTF liberates the sequence C-terminal of the GPS acting as an internal tethered agonist (TA) for the seven-transmembrane (7TM) domain [21]. CD97ΔNFT enhances Ga12/13-dependent activation of RHO [23,25]. It is not necessary that the NTF and CTF spatially dissociate; intact CD97 heterodimers exist at the cell surface. The core TA region becomes unmasked in the cleaved GAIN domain, and intra-GAIN domain movements regulate the level of TA exposure, thereby likely controlling aGPCR activity [26].

Next, the 7TM helical bundle permitting the regulation of the G protein signaling cascade follows.

The intracellular domain (ICD) of CD97, 46 amino acids in length, contains many phosphorylation sites (Figure 1a). The last two are part of the PDZ-binding motif (PBM) at the extreme C-terminus [13]. PDZ is a structural domain composed of 80–90 amino acids in scaffold proteins, which anchor transmembrane receptors to the cytoskeleton and hold together signaling complexes [27]. Phosphorylation of CD97(EGF1-5) at S833 (S740 in EGF125) is rapidly induced by mechanical stimuli and disrupts binding of the scaffold protein DLG1 to PDZ domains [25]. The PBM is critical for CD97-modulated detachment and mechanical cellular properties.

### 2.2. Multifaceted CD97 Interactions

The diverse array of interaction partners binding to or heterodimerizing with CD97 are described in the following section, starting with the ECD. Overall, CD97 lacks identified activating ligands.

There are four known interaction partners binding to the ECD (Figure 1c), none of which induce G protein signaling. CD55 [28] and Thy-1/CD90 [29], two glycophosphatidylinositol (GPI)-anchored transmembrane receptors, as well as chondroitin sulfate B [30], part of the proteoglycans attached to proteins, mediate adhesion between immune cells or of immune cells with (activated) endothelial cells.

Whether CD97 on tumor cells binds these interaction partners is unknown. CD97 and CD55 are co-expressed in several tumor entities [31,32,33,34,35,36,37], but the functional relevance of this relation has not been adequately clarified [38]. In contrast, the significance of the CD97–CD55 interaction in regulating immune functions has been convincingly shown [39] and has been receiving scientific attention again recently [40,41]. In mice, CD97 is downregulated on circulating leukocytes by CD55 [42], a process promoting splenic dendritic cell homeostasis [43]. Whether homing/adhesion of circulating tumor cells can be mediated by such a mechanism is an open question. The CD97-binding partner Thy-1 is also involved in cancer [44]. Thy-1 expression was reported in several tumor types, including liver, myeloid, skin, and brain tumors. Thy-1 is a cancer stem cell marker and, like CD97, regulates tumor migration, invasion, and metastasis. However, it is unknown whether, in tumors, CD97 indeed interacts with Thy-1 on the cells present there, such as the tumor cells themselves, (activated) endothelial cells, or fibroblasts.

The fourth CD97 ECD interaction partner are integrins. In an experimental in vitro and in vivo study, recombinant soluble CD97, that is the NTF, promoted tumor angiogenesis, whereby strong interactions of α5β1 and weaker interactions of αvβ3 integrin at endothelial cells with CD97 contributed [18].

CD97 heterodimerizes with the GPCR lysophosphatidic acid receptor 1 (LPAR1) at tumor cells, amplifying LPA-dependent signaling via Gα12/13 to RHO [23,45,46]. Targets of this pathway induce rearrangements of the (actin) cytoskeleton, necessary in various aspects of tumor cell biology, such as migration and invasion.

In (intestinal) epithelial cells, CD97 localizes to E-cadherin-based adherens junctions [47,48], a cell–cell contact transmitting signals to the actin cytoskeleton. In these hubs, β-catenin binds to the CD97 7TM/ICD, an interaction that is lost during colorectal tumorigenesis [49]. Ectopic CD97 strengthens the adherens junctions of enterocytes in mice by upregulating membrane-bound β-catenin, thus attenuating induced colitis [47].

aGPCRs can be activated by mechanical forces [50,51,52,53,54]. In CD97, mechanical stimuli induce phosphorylation at the last (and very likely at the second to last) serine located in the PBM. Phosphorylation or de-phosphorylation is the on/off mechanism for binding PDZ-domain-containing scaffold proteins to this motif. DLG1 is such an adaptor protein for CD97 [25].

### 2.3. Not Everywhere: ADGRE5 in Normal Tissues

CD97 is the only ADGRE family member not restricted to immune cells, as indicated by bulk RNA sequencing (RNAseq) data [55]. However, single cell (sc)RNAseq data reveal that in all tissues, the resident or infiltrated bone marrow-derived immune cells are those with the highest *ADGRE5* (The Human Protein Atlas, proteinatlas.org; accessed on 7 February 2022) (Figure 1d) [56]. A few tissue-specific cell types show low to moderate *ADGRE5* levels. This concerns smooth and skeletal muscle cells [16,17], alveolar type 1 (AT1) and AT2 [57], respiratory ciliated, gastric mucus secreting, and extravillus placental trophoblast cells. Nearly all organs fibroblasts, if present, had low *ADGRE5*, and the protein was yet not located in these cells. In summary, the bulk *ADGRE5* (sc)RNA data of normal, and likely also of tumor tissues, mainly represent *ADGRE5* in immune cells.

## 3. CD97 in Tumors

### 3.1. Most Tumor-Derived Cell Lines Are ADGRE5-Positive

**Figure 2:**
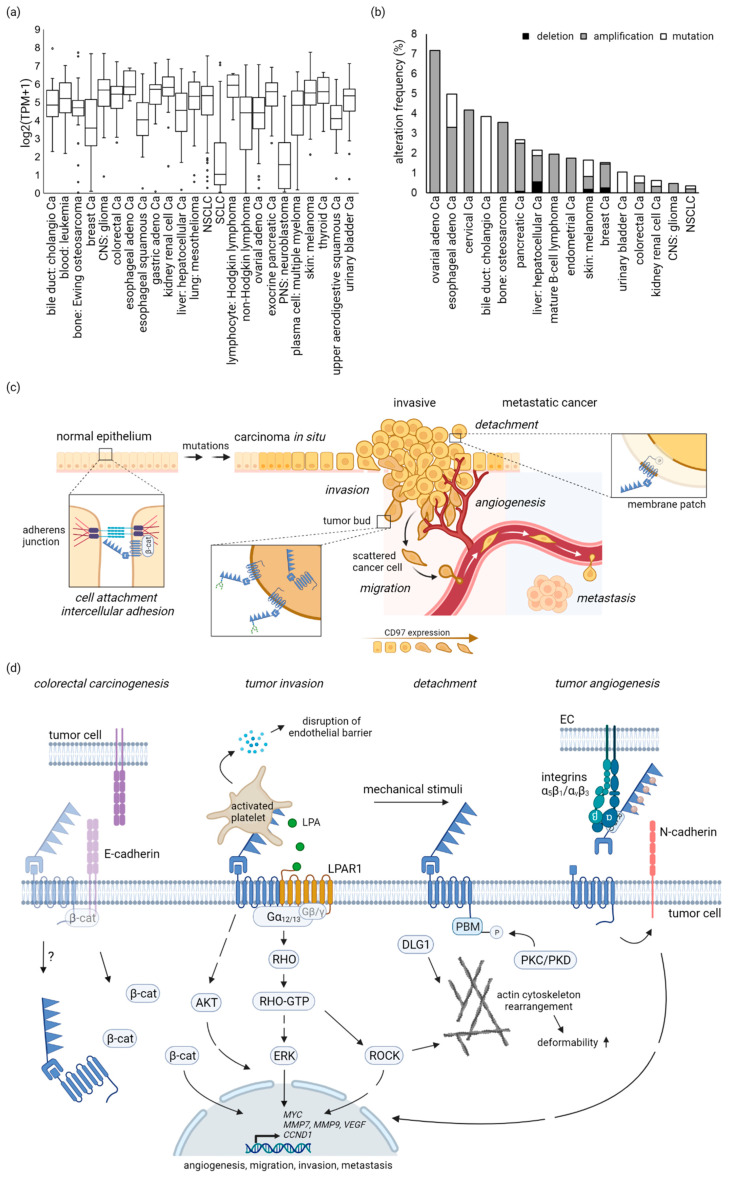
**CD97/*ADGRE5* in human cancer.** (**a**) ***ADGRE5* levels in tumor-derived cell lines**, grouped by lineage-derivation, in part with subtypes such as lung cell lines (Cancer Cell Line Encyclopedia, depmap.org; accessed on 15 February 2022). Except for small cell lung carcinomas (SCLCs, neuroendocrine tumors) and neuroblastoma, a malignant pediatric tumor of the peripheral (sympathetic) nerve system (PNS), most cell lines have moderate to high *ADGRE5*. Log2(TPM + 1) values are given. Ca (carcinoma); central nervous system (CNS); none small cell lung carcinoma (NSCLC) (**b**) **Frequency of *ADGRE5* gene alterations,** including somatic mutations, gene amplification, and deletion among various cancer (sub)types. The mutation frequency of *ADGRE5* in cancer is low (cBioPortal for Cancer Genomics, cbioportal.org; accessed on 28 March 2022; PanCancer Studies, *n* = 76,639 samples). (**c**) **CD97 localization and functions in tumors.** In normal epithelium, as in enterocytes, CD97 localizes to E-cadherin-based adherens junctions (left, insert), likely maintaining *intercellular adhesion*, whereas in (colorectal) tumor cells, it frequently disappears from these cell contacts and accumulates inside the cells (middle, insert), where its function is not clarified. In several tumor entities, such as colorectal, gastric, and gall bladder carcinoma, CD97 is enhanced in tumor buds, appearing as scattered tumor cells in histological sections, and/or in cells at the tumor invasion front compared with cells in the tumor center, indicating a key role of CD97 in *tumor invasion*. Mechano-dependent phosphorylation at the CD97 PBM modulates *cellular detachment*. pCD97 appears in situ in scattered colorectal tumor cells and leukocytes, i.e., cells that dissociate from other cells or from the ECM during migration and invasion. Detachment likely occurs intracellularly at the PBM, not at the ECD, as indicated by lost membrane patches of detaching cells seen in vitro (right insert). (**d**) **CD97-regulated signaling cascades and *functions* in tumors.** CD97 is involved in *epithelial-mesenchymal transition (EMT).* In colorectal cancers, junctional proteins such as E-cadherin, β-catenin (β-cat), and CD97 frequently disappear from adherens junctions. β-catenin emerges in the cytoplasm and translocates into the nuclei, now acting as a transcriptional co-activator driving carcinogenesis. *Tumor migration and invasion.* CD97 heterodimerizes and functionally synergizes with LPAR1 to promote tumor cell (transendothelial) migration and invasion. The association activates the heterotrimeric G-protein Gα12/13. Upon GDP-GTP exchange, this complex dissociates into Gα12/13 and Gβ/γ subunits. The Gα12/13 subunit activates RHO; thus, stimulating various downstream signaling molecules (e.g., ERK/AKT and ROCK), to finally result in tumor cell migration and invasion. In one scenario, activated platelets release dense granules, causing disruption of the endothelial barrier-enabling tumor cell extravasation and metastasis. *Cell detachment.* Mechanical forces induce phosphorylation of CD97 by protein kinase C (PKC) and/or D (PKD) at its intracellular PBM, disrupting CD97 binding to the scaffold proteins such as DLG1. In parallel, the actin cytoskeleton is modulated and cells detach, which is necessary for enhanced tumor cell migration and invasion. *Tumor angiogenesis.* In experimental studies, CD97 interacts with integrin on ECs via its RGD motif and the EGF-like repeats to promote the angiogenesis associated with tumor progression and inflammation, and modulates angiogenesis through upregulation of MMP-9 by inducing N-cadherin expression. The figure was created with BioRender.com (accessed on 26 April 2022).

The majority of tumor cell lines (Cancer Cell Line Encyclopedia, broadinstitute.org; accessed on 15 February 2022) have moderate to high *ADGRE5* levels (Figure 2a), indicating comparable *ADGRE5* levels in the parental tumors. However, it is also possible that *ADGRE5* was induced, or that single tumor cells with high *ADGRE5* were selected during the generation of the cell line and its adaption to monolayer culture.

Only 6.4% of the analyzed 1389 tumor cell lines had log2(TPM + 1) values ≤1. Among lung cell lines, the *ADGRE5* level discriminates between small cell lung cancer (SCLC)-derived cell lines, many with no or low *ADGRE5*, and non-small cell lung cancer (NSCLC)-derived cell lines (Figure 2a).

### 3.2. No Enrichment of Cancer-Related ADGRE5 Mutations

CD97/*ADGRE5* is induced or noticeably upregulated in many cancers (Tables S1–S3), whereas the corresponding normal tissue-specific cells have little or no *ADGRE5* [56]. The mechanisms leading to the (post)transcriptional CD97/*ADGRE5* induction or upregulation have not yet been characterized.

Genes can be affected by somatic mutations, copy number alterations (CNA), mutations in noncoding regions, dysregulation of microRNA (miRNA), epigenetic changes, and mutations in chromatin modifiers. The frequency of somatic *ADGRE5* mutations, verified in large cohorts, such as The Cancer Genome Atlas (TCGA), the Cancer Genome Project (CGP), and the International Cancer Genome Consortium (ICGC), and, thus, their impact on cancer is very low (cBioPortal for Cancer Genomics, cbioportal.org; accessed on 28 March 2022) (Figure 2b). For example, although *ADGRE5* is the most-mutated aGPCR gene in hepatocellular carcinoma, mutations only occur in 2% of cases [58].

Somatically acquired CNA, comprising deletions or amplifications of DNA fragments ≥1 kb, are particularly common in cancer and make a major contribution to its development and progression [59]. However, CNAs cannot explain the altered aGPCR mRNA expression in cancer [60]. Here, *ADGRE5* CNAs are not enriched [58].

### 3.3. Dysregulation of miRNAs and Epigenetic Changes Targeting ADGRE5 in Tumors

Dysregulation of miRNAs, which regulate genes at the posttranscriptional level by binding to the 3′-UTR of their target mRNA and repressing protein production by destabilizing this mRNA and translational silencing, has rarely been shown for CD97/*ADGRE5*. *ADGRE5* is a direct target of the tumor suppressor miR-126, often downregulated in tumors. Ectopic miRNA-126 decreases CD97 by binding to its 3′-UTR in breast cancer MDA-MB-231 cells [61]. miR-503-5p [62] and, as listed in UCSC Genome Browser (genome.ucsc.edu; accessed on 24 March 2022), miR-320, miR-21-5p/590-5p, and miR-505-3p.1 also control *ADGRE5* expression by targeting this region.

miRNA editing, a posttranscriptional mechanism introducing nucleotide changes, directs miRNAs to new target genes. Several miRNA editing events are critical regulators of tumor development. *ADGRE5* is a target gene of adenosine to inosine (A-to-I) edited miR-379-5p [63]. The editing level of miR-379-5p correlates directly to patients’ cancer-specific survival (TCGA). Edited miR-379-5p inhibits proliferation by promoting apoptosis in vitro [63]. It confers its phenotype through the downregulation of *ADGRE5.*

*ADGRE5* is also affected by epigenetic changes. In a genome-wide methylation study of esophageal carcinomas, *ADGRE5* was identified as one out of 23 (1/23) genes with relevance in tumor progression/metastasis [64]. Here, *ADGRE5* shows promoter hypomethylation and mRNA upregulation. *ADGRE5* is one of four highly methylated genes associated with poor progression-free survival in ovarian cancer, as seen in an integrated analysis of methylomic and genomic datasets (TCGA) [65].

### 3.4. Present in Body Fluids of Tumor Patients: Soluble CD97 (sCD97)

A direct consequence of the previously mentioned aGPCR GAIN domain cleavage is the release of the NTF from the receptor heterodimer. The NTF can be dislocated by means of receptor ligation or shedding, which likely requires an independent proteolytic cleavage event [66]. The CD97 ECD has no further cleavage site, except the GPS. Whether native sCD97 in body fluids includes the NTF remains unclear. Overall, sCD97 detection is related to malignant tumors and/or inflammation (Table S4). Inconsistent results on the detection of circulating sCD97 in patients with solid tumors [67,68] may be caused by the applied self-made ELISAs, which are not well-validated; spike-and-recovery and linearity-of-dilution experiments have not been performed when applying the various body fluids.

Theoretically, circulating sCD97 could engage with interactors over large distances and may be involved in paraneoplastic syndromes of tumor patients. In experimental in vivo studies, the CD97 NTF stimulated tumor angiogenesis through binding to integrins on endothelial cells [18]. In vitro, the NTF increased endothelial cell motility and invasion in an isoform-specific manner. Consequently, sCD97 from the various isoforms may cause different (patho)physiological effects by engaging specific ligands.

## 4. CD97 in Specific Tumor Entities

Here, we summarize the established links between CD97 and certain tumor entities. Tables S1–S3 contain details such as the methods, number of patients and controls, and results of each published study.

### 4.1. CD97 in Carcinomas

In CD97-targeted analyses, samples of solid tumors and the adjacent/corresponding normal tissue, ideally from the same patient, are compared using immunotechniques with CD97 antibodies. Immunohistology enables to type and quantify CD97-positive cells and to localize the receptor subcellularly, but it is time-consuming and subjective. In some studies, insufficient sample numbers were examined and (new) antibodies were often not validated for CD97-specificity and/or their suitability to stain paraffin-embedded tissue sections. Data obtained by hypothesis-free proteomic screening consistently support CD97-targeted analyses [61,69].

Furthermore, (re)analyses of transcriptomic and methylomic bulk tumor data were helpful to relate CD97 to cancer. Accordingly, *ADGRE5* is one of the most highly-expressed aGPCR across all cancers; e.g., in thyroid, gastric, and esophageal carcinomas and melanoma [58,60]. However, because of the extraordinarily high *ADGRE5* level in leukocytes, they reflect in part the (inflammatory) immune response directed to the tumor.

Table S1 contains the detailed published data on CD97 in carcinomas. In summary, CD97 protein is induced or significantly upregulated in esophageal, thyroid, hepatocellular, and intrahepatic cholangiocarcinoma, as well as in gall bladder, kidney, and prostate carcinoma [23,37,38,70,71,72]. CD97 is regulated at the (post)transcriptional level, since the corresponding normal cell types have little or no *ADGRE5* [56].

Interestingly, in some tumor entities, such as thyroid cancer, CD97 is induced in the most aggressive anaplastic carcinomas, but not, or rarely, in differentiated carcinomas [70,73], suggesting an important function of CD97 in invasion and metastasis. In some tumor entities, such as colorectal, gastric, and gall bladder carcinomas, CD97 is enhanced in tumor buds and/or in cells at the tumor cell margin compared with cells in the tumor center, further indicating a key role of CD97 in invasion [32,67,72] (Figure 2c). Tumor budding, the presence of isolated single scattered cancer cells or clusters of few cancer cells in histological sections, has prognostic value and is likely an independent predictive biomarker in solid cancers [74]. Computational simulation suggests that enhanced CD97 in tumor buds or at the tumor margin is regulated by the tumor microenvironment [75].

CD97 subcellular location changes during the transition to malignancy, suggesting new context-specific functions. Epithelial cells stick together by lateral junctions, in which transmembrane proteins, bridging the intercellular space, are anchored to the cytoskeleton by connecting proteins; ensuring tightening, mechanical stability, paracellular transport, and signal transduction. In tumor cells that have previously undergone (epi)genetic changes, epithelial-to-mesenchymal transition occurs, a process in which these junctions are disintegrated. Former junctional proteins accumulate inside the cells, acquiring new molecular interactions and, thus, functions. Exemplarily, in normal enterocytes, CD97 likely stabilizes adherens junctions by interacting with β-catenin [49], whereas in tumor cells it frequently disappears from these hubs and accumulates intracellularly, where its function remains unclear (Figure 2c,d). In parallel, β-catenin translocates into the nuclei [49], a hallmark of Wnt signaling in colorectal cancer [76,77]. Notably, CD97 is not a direct Wnt target gene [78].

### 4.2. CD97 in Primary Brain Tumors

*ADGRE5* is absent in nervous cells. Among the various glial cells, the supporting brain cells, only oligodentrocytes are slightly *ADGRE5*-positive [56]. Gliomas, which arise from glial cells, account for 78% of adult malignant brain tumors and show high molecular heterogeneity (WHO grades I–IV). Among these, glioblastoma multiforme (GBM, grade IV) is the most aggressive type of primary brain tumor; it pervasively infiltrates into the surrounding brain tissue. Thus, the current GBM treatment regimens are only marginally effective.

Importantly, *ADGRE5* in the affected cells conferred an invasive phenotype and poor survival in independent large cohorts of GBM patients (Table S2) [79,80,81]. CD97(EGF125) protein is present in GBM, but is absent or low in astrocytomas (WHO grade II and III). In vitro, CD97 knockout (CD97Ko) decreases migration and invasion of GBM cell lines and of glioma cells with stem cell properties [79]. CD97 is enriched in membrane fractions of invadopodia, actin-rich protrusions of invasive GBM cells [80]. Furthermore, glioblastomas display cellular hierarchies, with self-renewing glioma-initiating cells at the apex. CD97 has been identified as a potential biomarker for such glioma-initiating cells [82], a cancer population with neural stem cell properties, as well as tumor-initiating abilities and resistance to current therapies. Interestingly, Thy-1/CD90, one of the CD97 ECD binding partners, is considered a GBM stem cell marker, but it is also present in more differentiated GBM and many tumor-associated cells [44].

In summary, human studies and experimental approaches consistently indicate that CD97/*ADGRE5* represents a potential therapeutic target in GBM.

### 4.3. CD97 in Myosarcomas

Smooth and skeletal muscle cells have moderate *ADGRE5* [56]. The protein appears not only at the sarcolemma, but also intracellularly, at the longitudinal sarcoplasmatic reticulum of skeletal myofibres [17], where CD97 likely binds to or interacts with yet unknown proteins. In normal muscle cells, CD97 is non-glycosylated. It becomes *N*-glycosylated at the EGF-like repeats in many rhabdo- and leiomyosarcomas [16,17]. The latter are even more diverse: in one third of these tumors, CD97 is completely lost from sarcoma cells, which are still α-smooth muscle actin-positive.

### 4.4. CD97 in Leukemias

Circulating and tissue-resident or -infiltrating leukocytes have the highest *ADGRE5* levels among the more than 200 specific cell types in the human body [56]. Interestingly, CD97 was identified as a leukemia-associated marker by applying elaborate methods to high numbers of patients and healthy individuals (Table S3). (Re)analysis of genome-wide gene expression data of leukemic compared with bone-marrow (BM)-derived blasts of healthy individuals, and/or xenotransplantation of leukemic blasts into immunocompromised mice and subsequent transcriptomic/proteomic analysis of growing tumors, are first-line methodological approaches to select candidates involved in tumorigenesis [83,84]. Flow cytometry is the method of choice to validate these high-throughput data and to confirm candidates.

By correlating these data to the relevant clinic-pathological parameters of the patients, CD97/*ADGRE5* has been identified as a leukemia-associated candidate in acute myeloid (AML) [84], (pediatric) acute lymphoblastic [83,85], and chronic lymphocytic leukemia. CD97 is related to each patient’s subtype of specific leukemia, prognosis-relevant mutations, as in *FLT3*, and survival. In AML, CD97 is part of a signature of primary leukemic stem cell, a low-frequency leukemic subpopulation that possess stem cell properties and that is supposed to facilitate the development of relapse [84,86]. One of the main functions of increased CD97 expression in AML is to maintain an undifferentiated state. Thus, in AML, CD97 on leukemic stem cells is a promising therapeutic target. Furthermore, CD97 is a marker used in panels to monitor minimal residual disease in AML [87,88]

## 5. CD97-Regulated Signaling Cascades and Functions in Tumors

Experimental approaches elucidate which steps of tumorigenesis are regulated by CD97; details of the methods and results of each study are given in Table S5. Figure 2c,d visualize and summarize the main findings, while singular studies on specific topics are not taken into account.

Cells with ectopic CD97 or CD97Ko were applied to various in vitro functional assays or xenotransplanted into immunocompromised mice, in order to follow tumor growth and metastasis. Notably, in many experiments, CD97 downregulation was achieved using small hairpin RNA (shRNA), which decreased the protein only transiently in a part of the cells. Eventually, cells with partial CD97Ko were applied in functional assays.

Few data indicate that CD97 enhances proliferation and/or inhibits apoptosis of tumor cells [63,86,89]. Remarkably, although dysregulation of proliferation and/or cell death are key features of malignant tumors, most in vitro studies do not report data on either topic when investigating CD97-dependent cellular functions. Either these experiments were not realized, or, more likely, “negative” data were not published. Data from public databases (targetgenereg.org; accessed on 24 March 2022) report consistently that the variation in *ADGRE5* transcript expression is correlated to the cell cycle. *ADGRE5* mRNA has a peak phase in G2/M, which is two-fold higher compared with S-phase.

Most data characterize CD97 as a regulator of adherence/detaching, migration, invasion, and metastasis (Figure 2c). Immunohistological findings in humans support the experimental data: CD97 is especially induced in pervasive invasive tumors, such as GBM and anaplastic thyroid carcinoma [70,73], and is increased in tumor cell buds at the invasion front or in cells at the tumor margin of several different carcinomas [32,67,72].

CD97 is not only induced in tumors, but also undergoes changes in its (sub)cellular location, biochemical structure and/or expression level, resulting in new or altered cellular functions in various malignant tumors, arising from normal CD97-positive cells, such as enterocytes, myocytes, and leukocytes. Exemplarily, CD97 is lost at the adherens junctions of normal enterocytes during epithelial-to-mesenchymal transition, and appears intracellularly in colorectal tumor cells [49] (Figure 2c,d). Notably, in a few cases, CD97 is markedly reduced or even vanishes from tumors in which the respective normal cell type express CD97, such as in leiomyosarcomas, the malignant tumors of smooth muscle [16].

The high number of human studies analyzing CD97 in malignant tumors and relating these data to clinical patients’ parameters contrast with the low number of studies investigating CD97-signaling in cancer. We found several CD97-regulated signaling scenarios (Figure 2d).

CD97 can use GPCR-mediated signaling pathways transducing extracellular signals to G proteins and small GTPases. Ectopic CD97 without the NTF (ΔNTF) amplifies serum response element (SRE)-dependent signaling, indicating Gα12/13-dependent RHO activation [23,25,45]. Neither other G-proteins nor CREB, NFAT, or NFκB are involved in CD97-regulated signal transduction [25].

Heterodimerization of CD97 with LPAR1 partly proceeds Gα12/13-dependent signaling. In this way, activation of platelets by CD97 on tumor cells leads to a situation in which LPA is released from the platelets, giving the tumor cell more invasive properties, as a result of binding to a CD97-LPA receptor complex [46]. Interestingly, RHO proteins can interact with a considerable number of targets, directly affecting cellular contractility, motility, and migration, and (actin) cytoskeleton rearrangement, which is in line with CD97-regulated cellular functions in normal and tumor cells. For example, CD97Ko clones of MDA-MB-231 breast cells changed their F-actin structure under shear stress and had an altered deformability in an optical stretcher compared with wild-type cells [25].

Infrequently, CD97 is associated with regulation of the mitogen-activated protein kinase/extracellular signal-regulated kinase (MAPK/ERK) and phosphatidylinositol 3-kinase/protein kinase B (PI3K/AKT) pathways, either in normal settings [42,47] or in experimental cancer studies. CD97Ko in prostate DU145 cancer cells downregulated phospho-ERK (pERK) and pAKT [23]. AKT activation is also decreased in GBM cell lines after CD97Ko [90]. Consistently, transgenic *Adgre5* increased the frequency of pERK- and Ki67-positive tumor cells in a thyroid cancer model [45]. However, the data situation is unconvincing, as only Western blot analyses of single samples are available.

CD97 modulates cellular detachment. Mechanical forces lead to Ser833 phosphorylation in the CD97 PBM, uncoupling it from PDZ-domain-containing scaffold proteins. The consequence is that cortical F-actin depolymerizes and the cells detach. Whether CD97 receives and transmits the mechanical stimuli itself and how actin and CD97 interconnect remains unclear. Interestingly, tumor cells located at the invasion front of a colorectal carcinoma and several leukocytes in normal spleen and in ectopic lymphoid follicles were pCD97-positive, indicating detachment in vivo [25].

## 6. Summary and Future Challenges

Clinical data reveal that CD97 is certainly relevant in tumorigenesis. CD97 is induced in many tumor entities in which the corresponding tissue-specific normal cells have no or low *ADGRE5*. In tumors derived from normal cells with moderate to high CD97/*ADGRE5*, CD97 undergoes changes in its subcellular location, glycosylation state, and/or expression during tumorigenesis, indicating new tumor-specific CD97 functions.

Among the various hallmarks of cancer [91], invasion and metastasis are the most deleterious in terms of clinical outcome [92] and the ones in which CD97 definitely plays a role, as seen in many valuable clinical, as well as in vivo and in vitro, experimental studies. The role of CD97 in the cancer hallmarks “resisting cell death” and “inducing (tumor) angiogenesis” has infrequently been experimentally shown; these data await (clinical) confirmation.

CD97 enhances Gα12/13-dependent activation of RHO. Its targets affect cellular contractility, migration/motility, or cytoskeleton rearrangement, consistently with the function of CD97 in cancer. However, the single tumor-specific signaling pathways and targets have barely been characterized. Future studies need to define the detailed CD97-regulated signaling steps, the CD97 ligands/interacting partners involved, and the isoforms in specific, stage-dependent tumor entities.

In summary, CD97 is a potential target for tumor therapy. However, although it is obviously induced or altered in many tumor entities and is a marker of tumor-initiating/tumor stem cells in GBM and AML, only one first-stage trial has addressed CD97-directed treatment. To target CD97-dependent microvascular invasion in hepatocellular carcinoma, a drug-loaded CD97-specific nanocomposite platform was tested for biocompatibility and reduced damage of normal tissues [41]. The reason for the reluctance to target CD97/*ADGRE5* in tumors is its high level in normal leukocytes, rendering systemic targeting of CD97 in tumors difficult or even impossible. CD97-directed therapy would also target natural and therapeutically-induced anti-tumor immune responses, which require the peripheral immune system [93]. In GBM, the blood–brain barrier is an additional difficulty that hinders therapeutic agents from reaching glioblastoma tissues. Likely, the identification of CD97-regulated targets for which therapeutic intervention can be devised and the development of CD97-specific agonists and antagonists will open prospective therapeutic approaches for this receptor.

## Data Availability

Publicly available datasets were reanalyzed in this study. This data can be found here: https://www.proteinatlas.org/ENSG00000123146-ADGRE5/single+cell+type (accessed on 7 February 2022); https://depmap.org/portal/gene/ADGRE5?tab=characterization (accessed on 15 February 2022); cBioPortal for Cancer Genomics: ADGRE5 in MSK-IMPACT Clinical Sequencing Cohort (MSKCC, Nat Med 2017) and nine other studies https://www.cbioportal.org/results/cancerTypesSummary?cancer_study_list=msk_ch_2020%2Cpan_origimed_2020%2Cmsk_met_2021%2Cmsk_impact_2017%2Cmixed_allen_2018%2Cmetastatic_solid_tumors_mich_2017%2Cpancan_pcawg_2020%2Csummit_2018%2Ctmb_mskcc_2018%2Cntrk_msk_2019&Z_SCORE_THRESHOLD=2.0&RPPA_SCORE_THRESHOLD=2.0&profileFilter=mutations%2Cfusion%2Ccna%2Cgistic&case_set_id=all&gene_list=ADGRE5&geneset_list=%20&tab_index=tab_visualize&Action=Submit (accessed on 28 March 2022).

## Supplementary Materials

The following supporting information can be downloaded at https://www.mdpi.com/article/10.3390/cells11091538/s1: Table S1: Expression of CD97 in carcinomas; Table S2: CD97 in GBMs; Table S3: CD97 in hematopoietic malignancies; Table S4: sCD97 in human body fluids; Table S5: CD97-dependent cellular functions in tumors. Key studies are highlighted in the References. See also: http://www.ensembl.org/Homo_sapiens/Gene/Summary?db=core;g=ENSG00000123146;r=19:14380501-14408725, accessed on 4 February 2022; https://www.phosphosite.org/uniprotAccAction?id=P48960, accessed on 4 February 2022; http://wormweb.org/exonintron, accessed on 15 March 2022; https://genome.ucsc.edu/cgi-bin/hgTracks?db=hg19&lastVirtModeType=default&lastVirtModeExtraState=&virtModeType=default&virtMode=0&nonVirtPosition=&position=chr19%3A14485061%2D14526433&hgsid=1345666819_QwPMXzTjY20D4atEZ3SlfelPgOUR, accessed on 24 March 2022; www.targetgenereg.org, accessed on 24 March 2022.

## Abbreviations

AML	acute myeloid leukemia
aGPCR	adhesion G protein-coupled receptor
CD97Ko	CD97 knockout
CNA	copy number alteration
CTF	C-terminal fragment
ECD	extracellular domain
ECM	extracellular matrix
GBM	glioblastoma multiforme
GAIN	GPCR autoproteolysis-inducing
GPS	GPCR proteolytic site
ICD	intracellular domain
miRNA, miR	micro RNA
NTF	N-terminal fragment
PBM	PDZ-binding motif
7TM	seven-transmembrane
sCD97	soluble CD97
TA	tethered agonist
TCGA	The Cancer Genome Atlas
