# Supplementary material for: To Detach, Migrate, Adhere, and Metastasize: CD97/ADGRE5 in Cancer
Source: Cells. 2022 May 4;11(9):1538. doi: 10.3390/cells11091538 (PMC9101421; doi:10.3390/cells11091538)
Supplement: Supplementary file 1 [file cells-11-01538-s001.zip › cells-1691515-supplementary.pdf]

To detach, migrate, adhere, invade and metastasize: CD97/*ADGRE5* in cancer

Supplementary Materials

Table S1. Expression of CD97 in carcinomas

**tissue**	**tumor**	**literature**	**method**	**result, conclusion**
esophagus	esophageal Ca	[1]	IH CD97 Ca vs adjacent normal (n=13 patients)	10/13 Ca CD97+
		[2]	genome-wide methylation study (epigenomics)	*ADGRE5* is 1/23 genes with relevance in tumor progression/metastasis
stomach	gastric Ca	[1]	IH CD97 Ca vs adjacent normal (n=50 patients)	44/50 Ca CD97+
		[3]	IH CD97 Ca vs adjacent normal (n=35 patients)	high CD97 at tumor margin, role of CD97 in tumor invasion
intestine	colorectal Ca	[4]	IH CD97 Ca vs adjacent normal (n=81 patients)	↑CD97 in Ca; 75/81 Ca CD97+; CD97 enhanced at the invasion front
	rectal Ca	[5]	IH CD97 Ca vs adjacent normal (n= 71 patients)	↑CD97 in Ca, high CD97 at invasion front is associated with poor survival
liver	HCC	[6]	IH CD97 Ca vs adjacent normal (n=140)	↑CD97 in HCCs is positively correlated with poor prognosis and negatively correlated with GRK6
		[6]	*in vivo* xenograft model using Huh7 cells with ectopic CD97	ectopic CD97 ↑lung metastasis
		[6]	Huh7 and SMMC-7721 cells with ectopic CD97 or GRK6, *in vitro*	ectopic CD97 ↑MMP2 and MMP9 secretion in the absence of GRK6
	cholangio Ca (intrahepatic)	[7]	IH CD97; bile sCD97 ELISA (n=71 Ca vs n=10 hepatolithiasis patients)	54/71 Ca CD97+, CD97 and sCD97 independent risk factor for survival
gall bladder	gall bladder Ca	[8]	IH CD97 Ca vs adjacent normal (n=138 patients)	96/138 Ca CD97+, enhanced at invasive front; CD97 independent risk factor for overall survival
pancreas	PDAC	[9]	IH(P) CD97 (n= 50 Ca, n=36 normal pancreatic), but the used CD97 Ab is not approved for paraffin	6/50 Ca CD97+, only poorly differentiated PDCA
		[1]	IH(cryo) CD97 (n=18 patients)	14/18 Ca CD97+
		[10]	IH CD97 vs adjacent normal (n=37 patients); CD97 Ab: polyclonal, Santa Cruz	↑CD97 in Ca vs. normal, 37/37 Ca CD97+, CD97 is related to invasion/metastasis and prognosis
kidney	ccRCC	[11]	urinary N-glycoproteome (n = 15 pT1, n = 15 pT3 patients, n=15 controls)	CD97 is 1/3 glycoproteins ↑ in urine of patients with higher pT
thyroid	thyroid Ca	[12]	IH CD97 (n=10 differentiated, n=12 undifferentiated; n=11 normal thyroid)	CD97 induction in Ca; ↑CD97 correlates to dedifferentiation
		[13]	IH CD97 (n=29 patients)	CD97 correlates to pTNM
			in vitro CD97 regulation by growth factors in FTC-133 cells	EGF ↑number of CD97+ cells, retinoic acid ↓number of CD97+ cells
		[14]	qRT-PCR, treatment thyroid Ca cell lines	nutritional (beneficial) polyphenol phytochemicals ↓*ADGRE5*
		[15]	IH CD97 tissue array	percentage of CD97+ tumors higher in poorly/undifferentiated vs papillary/follicular Ca
		[15]	*in vivo*: thyroglobulin- promotor driven *Adgre5* combined with	*Thrb*$^{PV/PV}$ mice: CD97 induction in Ca; *Thrb*$^{PV/PV}$ x *Adgre5*: ↑vascular invasion and lung metastasis

			Thrb^{PV} (thyroid follicular carcinogenesis), mice	
	medullary thyroid Ca	[16]	IH CD97, qRT-PCR (n=54 patients)	*ADGRE5* correlates to pT stage
prostate	prostate Ca	[17]	IH CD97 tissue array, qRT-PCR	↑CD97 in prostatic intraepithelial neoplasia, primary and metastatic prostate cancer vs normal
	prostate adeno Ca	[18]	IH CD97, tissue array (n=68 Ca, n=15 normal)	normal CD97-, 59% Ca CD97+; CD97 and LPAR1 co-expressed in Ca
		[18]	CD97ko (shRNA) in DU145 cells, transwell chamber invasion	CD97ko ↓invasion (LPAR1-dependent) and ↓LPA-dependent signaling to RHO and ERK activation
		[18]	*in vivo* xenograft model using CD97Ko PC3 cells	↓bone metastasis, unchanged sc tumor growth
ovary	ovarian high-grade serous Ca	[19]	proteomes[#] of paired primary and recurrent post-chemotherapy Ca (n=9)	CD97 is one protein ↑ in recurrent compared with primary tumors
	ovarian Ca	[20]	*in vivo* xenograft model of SKOV3 or OVCAR3 cells pre-enriched for stem-like cells	CD97 is 1/5 CD markers ↑ in cancer stem-like vs. parental cells
		[21]	methylomics and genomics datasets; TCGA (n= 391 patients)	*ADGRE5* is 1/4 highly methylated genes associated with poor progression-free survival (unclear whether tumor and/or immune cells)
		[22]	*in vitro* cancer cell lines; tumor transendothelial migration assay, medium BME cell invasion assay, stimulation with recombinant hCD55	LPS or paclitaxel-resistance ↑CD97 through ↓miR-503-5p (targeting *ADGRE5* 3'-UTR); ↑migration and invasion
breast	breast Ca	[23]	*in vitro,* miR-126 overexpression in MDA-MB-231 cells, proteome	CD97 is a direct target of tumor suppressor miR-126, which is often ↓in tumors
oral cavity	OSCC	[24]	IH CD97, qRT-PCR (n=78 OSCC, n=10 normal oral mucosa)	CD97 is a marker of dedifferentiated OSCC; normal oral mucosa: basal layer CD97+

Ca carcinoma, ccRCC clear cell renal cell Ca, HCC hepatocellular carcinoma, IH immunohistology, IH(C) cryosections, IH(P) paraffin-embedded, OSCC oral squamous cell carcinoma, PDCA pancreatic ductal adenocarcinoma

[#]CIEF/Nano-RPLC: papillary isoelectric focusing/nano-reversed phase liquid chromatography

[##]combined BONCAT (bioorthogonal non-canonical amino acid tagging) and SILAC (stable isotope labeling by amino acid in cell culture)

Table S2. CD97 in GBMs

literature	method	result/conclusion
[25]	*in vitro*, transcriptome after WT1Ko (siRNA) in GBM cell lines	WT1Ko ↓invasiveness and ↓*ADGRE5*
[26]	*in vitro*, transcriptome after CD97Ko (siRNA) in GBM cell lines	CD97Ko ↓migration and invasion; proliferation unchanged
[26]	transcriptome GBM cohort (TCGA n= 539 patients)	*ADGRE5* has prognostic significance
[27]	GBM xenograft model plus iv. delivered phage peptide library; tumor-derived GICs are analyzed for bound peptides	CD97 is a potential biomarker for GICs
[28]	*in vitro*, invadopodia assay (9 GBM cell lines), MS-based proteomic analyses	CD97 is enriched in invadopodia
[28]	transcriptome of GBM cohorts (TCGA n=539, REMBRANDT n= 187 patients)	*ADGRE5* has prognostic significance
[29]	WB, qRT-PCR in glioma samples	CD97 present in GBMs but not astrocytomas (WHO II, III)
[29]	transcriptome GBM cohort (TCGA)	↑*ADGRE5* in classical/mesenchymal, not neural/proneural GBM subtypes
[30]	*in vitro*, primary patient-derived glioma stem cells (n=5 patients), manipulation CD97 level; correlation to clinic	CD97Ko ↓invasion rate, CD97 overexpression ↑invasion rate
[31]	CD97Ko (siRNA) in GBM cell lines, screening for CD97-regulated pathways	CD97Ko ↓PI3K/AKT and MAPK/ERK signaling (transcriptomic data)<br>CD97Ko ↓AKT activation (WB)

GIC glioma-initiating cell, MS mass-spectrometry, REMBRANDT Repository of Molecular Brain Neoplasia Data, TCGA The Cancer Genome Atlas, WB Western Blot analysis, WT1 Wilms tumor 1

Table S3. CD97 in hematopoietic malignancies

leukemia	literature	methods, number of patients/controls	results/conclusion
**ALL**	[32]	mRNA microarray of lymphoblasts (n= 270 childhood ALL) to normal B-cell progenitors	CD97 is 1/30 genes differentially expressed by > 3-fold in at least 25% of ALL cases
	[32]	conformation by flow cytometry (n= 200 B-lineage ALL, n= 61 nonleukemic BM)	CD97 is 1/22 antigens differentially expressed in up to 81.4% of ALL, especially in hyperdiploid
BCP-ALL	[33]	leukemia xenograft model (n=19 patients); analysis surface proteome by Cell Surface Capture (CSC) technology	CD97 is a (new) leukemia-associated marker
	[33]	conformation by flow cytometry (a.o. n=86 patients)	CD97 is 1/4 CD antigens accounting for the most informative differences between normal and malignant cells
	[34]	flow cytometry (n=84 B-lineage ALL, n=15 controls/ B cell precursors)	CD97 is part of a panel to detect minimal residual disease in childhood ALL
	[35]	mRNA microarray (n=5 BCP-AL; n=5 normal BM); flow cytometric analyses (n=63 BCP-ALL)	CD97 overexpression in pediatric ALL; CD97 not essential for ALL proliferation and engraftment
**AML**	[36]	leukemia xenograft model (n= 61 patients)	identification of a primary LSC gene signature
	[36]	transcriptomic analysis 2 independent array platforms, evaluation of candidates by flow cytometry	CD97 enriched in LSC compared with HSC
	[37]	proteomics by nano-LC-MS/MS, combined with transcriptomics	identification of CD97 to be associated with AML
	[38]	flow cytometry (n= primary 385 AML, n=10 normal BM, n=15 MDS)	↑CD97 in 208/285 AML, accompanied by ↑BM blast count and ↑*FLT3* mutations
	[39]	flow cytometry of LSC with defined markers (AML at diagnosis and relapse n=25 patients); leukemia xenograft model	no change of CD97 between diagnosis and relapse
	[40]	NGS transcriptome GPCRs (n=148 AML; normal blood and BM cell populations)	CD97 is 1/30 AML-overexpressed GPCRs
	[41]	transcriptome, genome wide gene expression (mRNA microarray) of AML cells (n=157 patients) with normal myeloblasts	CD97 is 1/22 markers aberrantly expressed in AML
	[41]	flow cytometry (n=240 AML patients); machine-learning algorithm	markers (including CD97) allow minimal residual disease (MRD) monitoring
	[42]	TCGA transcriptomic data re-analysis (n=173 AML)	high *ADGRE5* expression is associated with poor overall survival
	[43]	metaanalysis of transcriptomic data (gexc.riken.jp/), conformation by flow cytometry (n=30 patients) and other approaches	*ADGRE5* is overexpressed in LSC-enriched cells; CD97 is higher in enriched LSCs compared to HSCs; high levels of CD97 correlate with poor prognosis
	[43]	experimental: CD97Ko by shRNA in AML cell lines, xenotransplantation	CD97Ko ↓disease aggressiveness *in vivo*
	[43]	CD97Ko in AML cell lines (HL-60, U937)	CD97Ko ↑proliferation, ↓apoptosis, ↓AML blast differentiation
	[44]	transcriptomic data re-analysis only for aGPCRs	↑*ADGRE5* is associated with ↓overall survival
	[45]	TCGA transcriptomic data re-analysis (n=167 AML)	*ADGRE5* levels part of a mRNA and lncRNAs profile with a role in prognosis and risk stratification
	[46]	routine flow cytometry plus 7 potential AML markers (n=256 AML, n=11 normal BM)	CD97 part of a pipeline for diagnosis, MRD detection and clonal tracking AML
**CLL**	[47]	*in vitro*, CLL culture with fibroblasts; DotScan™ antibody microarray to identify changed CD antigens	CD97 is 1/25 changed CD antigens; ibrutinib or idelalisib countered the change of CD97

ALL acute lymphoblastic leukemia, AML acute myeloid leukemia, CLL chronic lymphocytic leukemia, BCP-ALL B-cell precursor ALL, BM bone marrow, CD cluster of differentiation, HSC hematopoietic stem cell, LC-MS/MS liquid chromatography/tandem mass spectrometry, lncRNA long non-coding RNA, LSC leukemic stem cells, MDR minimal residual disease, MDS myelodysplastic syndrome, NGS next generation sequencing, The Cancer Genome Atlas (TCGA)

Table S4. sCD97 in human body fluids

body fluid	literature	number patients, normals	method	CD97 Abs used	result
serum, plasma	[48]	normals	IP, WB	polyclonal Ab, N-terminus	sCD97-negative
synovial fluid	[48]	inflammatory arthritis (n=12)	IP, WB	polyclonal Ab, N-terminus	sCD97 in body fluids is related to inflammation
synovial fluid	[49]	rheumatoid arthritis (RA, n=30), osteoarthritis (OA, n=13), reactive arthritis (RA, n=10)	self-made sandwich ELISA	capture Ab: MEM-180 (GAIN); detection Ab CLB-CD97/1 (EGF-like)	sCD97 higher in RA than in OA and reactive arthritis
serum	[4]	colorectal cancer (n=81), normal (n=26)	self-made sandwich ELISA	as [49]	no difference between patients and normals
	[50]	colorectal (n=11), pancreatic (n=11), breast cancer (n=15), normal (n=30)	self-made sandwich ELISA	capture monoclonal Ab VIM3C (EGF-like), detection Ab 8-154	↑sCD97 in cancer patients
bile	[7]	intrahepatic cholangiocarcinoma (n=71), hepatolithiasis (n=10)	self-made sandwich ELISA	as [49]	sCD97 independent risk factor for survival, predicts lymph node metastasis
pleural effusion (PE)	[51]	malignant PE (n= 51), tuberculous PE (n=55)	commercial ELISA	unclear	sCD97 tuberculous > malignant PE

Ab antibody, WB Western Blotting, IP immunoprecipitation

Table S5. CD97-dependent cellular functions in tumors

cellular function	literature	methods	result
apoptosis	[52]	*in vitro* CD97Ko (lentiviral) and stable ectopic CD97 in fibrosarcoma HT1080 cells, apoptosis assays	CD97Ko ↑apoptosis, ectopic CD97 ↓apoptosis; depends on GPS-cleavage
apoptosis	[43]	CD97Ko (shRNA) in AML cell lines (HL-60, U937), apoptosis and proliferation assays	CD97Ko ↓proliferation by ↑apoptosis
stem cell maintenance	[43]	CD97Ko (shRNA) in AML cell lines (HL-60, MOLM13), quantitation myeloid markers	CD97Ko ↑AML blast differentiation
proliferation, apoptosis	[53]	edited miRNA-379-5p inhibits proliferation and promotes apoptosis, screening for target genes	*ADGRE5* is the a major target gene through which these functions of edited miRNA-379-5p are mediated
proliferation	[54]	CD97Ko in gastric SGC-7901 cells, isolation and application of exosomes	exosomes of WT, not CD97Ko cells ↑proliferation (activating MAPK signaling)
tumor growth	[55]	*scid* mouse model, xenotransplantation of fibrosarcoma HT1080 cells with ectopic CD97	CD97 accelerates initiation of tumor growth but not growth velocity; depends on CD97 EGF-like repeats and 7TM
angiogenesis	[56]	directed *in vivo* angiogenesis assay (DIVAA) in mice	NTF of CD97(125) promotes tumor angiogenesis
angiogenesis	[56]	subcutaneous tumor induction with ectopic CD97 NIH-3T3 cells in mice	CD97(EGF1-5) ↑vascularization of developing tumors
migration (directed)	[4]	*in vitro*, transwell chamber assay (serum); colorectal tumor cell lines (n=15)	migration correlates with the CD97 level at the cell surface
migration (non- directed)	[55]	*in vitro*, quantitation of covered distances, fibrosarcoma HT1080 cells stable overexpressing CD97	CD97 ↑single cell migration; depends on EGF-like repeats and 7TM
migration/ invasion	[57]	CD97 overexpression in BGC-823 gastric cancer cells	CD97(EGF125) ↑motile and invasive ability
migration/invasion (directed)	[18]	*in vitro*, transwell chamber assay (serum); CD97Ko (shRNA) in prostate DU145 cancer cells	CD97Ko ↓migration/invasion
migration/ invasion	[26]	*in vitro*, transcriptome after CD97Ko (siRNA) in GBM cell lines	CD97Ko ↓migration and invasion; proliferation unchanged
migration (directed)	[38]	*in vitro*, transwell chamber assay (serum and LPA); CD97Ko (shRNA) in AML MV4-11 cells	CD97Ko ↓migration/invasion
adhesion, migration, invasion/metastasis	[58]	a.o. breast Mvt-1 and prostate DU145/Ras cells (WT vs. CD97Ko, pretreated with platelets) xenotransplantation, lung and bone metastasis assay	platelets (activated by CD97 on tumor cells) release LPA which binds to CD97-LPAR complex at tumor cells; ↑metastasis
migration/invasion	[22]	ovarian cancer cell lines; tumor transendothelial migration assay, medium BME cell invasion assay, stimulation with recombinant hCD55	LPS-stimulation or paclitaxel-resistance ↑CD97 through ↓miR-503-5p
migration/ invasion	[6]	*in vitro*, wounding and transwell chamber assays; stable CD97Ko in HCC SMM-7721 cells, stable ectopic CD97 in HCC Huh-1 cells	CD97 ↑migration and invasion in presence of CD55
invasion	[28]	*in vitro*, invadopodia assay (9 GBM cell lines), MS-based proteomic analyses	CD97 is enriched in invadopodia
invasion	[30]	*in vitro*, primary patient-derived glioma stem cells (n=5 patients), manipulation CD97 level	CD97Ko ↓invasion rate, CD97 overexpression ↑invasion rate
detachment	[59]	fibrosarcoma HT1080 cells with ectopic CD97 or CD97ΔPBM, shear-stress application	CD97 regulates detachment via the PBM
deformability	[59]	CD97Ko (by CRISPR-Cas) in breast MDA-MB-321 cells, optical stretching of single cells	↑deformability in CD97Ko clones compared to WT

AML acute myeloblastic leukemia; HCC hepatocellular carcinoma, IH immunohistology, in situ (using human tissues)

References

1. Aust, G.; Steinert, M.; Schütz, A.; Wahlbuhl, M.; Hamann, J.; Wobus, M. CD97, but not its closely related EGF-TM7 family member EMR2, is expressed on gastric, pancreatic and esophageal carcinomas. *Am. J. Clin. Pathol.* **2002**, *118*, 699–707.
2. Singh, V.; Singh, L.C.; Vasudevan, M.; Chattopadhyay, I.; Borthakar, B.B.; Rai, A.K.; Phukan, R.K.; Sharma, J.; Mahanta, J.; Kataki, A.C.; et al. Esophageal cancer epigenomics and integrome analysis of genome-wide methylation and expression in high risk northeast indian population. *OMICS.* **2015**, *19*, 688–699.
3. Liu, Y.; Chen, L.; Peng, S.; Chen, Z.; Gimm, O.; Finke, R.; Hoang-Vu, C. The expression of CD97EGF and its ligand CD55 on marginal epithelium is related to higher stage and depth of tumor invasion of gastric carcinomas. *Oncol. Rep.* **2005**, *14*, 1413–1420.
4. Steinert, M.; Wobus, M.; Boltze, C.; Schütz, A.; Wahlbuhl, M.; Hamann, J.; Aust, G. Expression and regulation of CD97 in colorectal carcinoma cell lines and tumor tissues. *Am. J. Pathol.* **2002**, *161*, 1657–1667.
5. Han, S.L.; Xu, C.; Wu, X.L.; Li, J.L.; Liu, Z.; Zeng, Q.Q. The impact of expressions of CD97 and its ligand CD55 at the invasion front on prognosis of rectal adenocarcinoma. *Int. J. Colorectal Dis.* **2010**, *25*, 695–702.
6. Yin, Y.; Xu, X.; Tang, J.; Zhang, W.; Zhangyuan, G.; Ji, J.; Deng, L.; Lu, S.; Zhuo, H.; Sun, B. CD97 promotes tumor aggressiveness through the traditional G protein-coupled receptor-mediated signaling in hepatocellular carcinoma. *Hepatology* **2018**, *68*, 1865–1878.
7. Meng, Z.-W.; Liu, M.-C.; Hong, H.-J.; Du, Q.; Chen, Y.-L. Expression and prognostic value of soluble CD97 and its ligand CD55 in intrahepatic cholangiocarcinoma. *Tumour Biol.* **2017**, *39*, 1010428317694319, doi:10.1177/1010428317694319.
8. Wu, J.; Lei, L.; Wang, S.; Gu, D.; Zhang, J. Immunohistochemical expression and prognostic value of CD97 and its ligand CD55 in primary gallbladder carcinoma. *J. Biomed. Biotechnol.* **2012**, *2012*, 587672. doi: 10.1155/2012/587672, doi:10.1155/2012/587672.
9. Boltze, C.; Schneider-Stock, R.; Aust, G.; Dralle, H.; Roessner, A.; Hoang-Vu, C. Fas, Fas-L and CD97 as distinction markers between chronic pancreatitis and pancreatic ductal adenocarcinoma in perioperative evaluation of cryocut sections. *Exp.Clin.Endocrinol.Diab.* **2002**, *110 (suppl.1)*, 57.
10. He, Z.; Wu, H.; Jiao, Y.; Zheng, J. Expression and prognostic value of CD97 and its ligand CD55 in pancreatic cancer. *Oncol. Lett.* **2015**, *9*, 793–797.
11. Santorelli, L.; Capitoli, G.; Chinello, C.; Piga, I.; Clerici, F.; Denti, V.; Smith, A.; Grasso, A.; Raimondo, F.; Grasso, M.; et al. In-pepth mapping of the urinary N-glycoproteome: distinct signatures of ccRCC-related progression. *Cancers (Basel)* **2020**, *12*, doi:10.3390/cancers12010239.
12. Aust, G.; Eichler, W.; Laue, S.; Lehmann, I.; Heldin, N.-E.; Lotz, O.; Scherbaum, W.A.; Dralle, H.; Hoang-Vu, C. CD97: A dedifferentiation marker in human thyroid carcinomas. *Cancer Res.* **1997**, *57*, 1798–1806.
13. Hoang-Vu, C.; Bull, K.; Schwarz, I.; Krause, G.; Schmutzler, C.; Aust, G.; Köhrle, J.; Dralle, H. Regulation of CD97 protein in thyroid carcinoma. *J. Clin. Endocrinol. Metab.* **1999**, *84*, 1104–1109.
14. Kang, H.J.; Youn, Y.-K.; Hong, M.-K.; Kim, L.S. Antiproliferation and redifferentiation in thyroid cancer cell lines by polyphenol phytochemicals. *J. Korean Med. Sci.* **2011**, *26*, 893–899, doi:10.3346/jkms.2011.26.7.893.
15. Ward, Y.; Lake, R.; Martin, P.L.; Killian, K.; Salerno, P.; Wang, T.; Meltzer, P.; Merino, M.; Cheng, S.Y.; Santoro, M.; et al. CD97 amplifies LPA receptor signaling and promotes thyroid cancer progression in a mouse model. *Oncogene.* **2013**, *32*, 2726–2738.
16. Mustafa, T.; Klonisch, T.; Hombach-Klonisch, S.; Kehlen, A.; Schmutzler, C.; Koehrle, J.; Gimm, O.; Dralle, H.; Hoang-Vu, C. Expression of CD97 and CD55 in human medullary thyroid carcinomas. *Int. J. Oncol.* **2004**, *24*, 285–294.

17. Loberg, R.D.; Wojno, K.J.; Day, L.L.; Pienta, K.J. Analysis of membrane-bound complement regulatory proteins in prostate cancer. *Urology.* **2005**, *66*, 1321–1326.
18. Ward, Y.; Lake, R.; Yin, J.J.; Heger, C.D.; Raffeld, M.; Goldsmith, P.K.; Merino, M.; Kelly, K. LPA receptor heterodimerizes with CD97 to amplify LPA-initiated RHO-dependent signaling and invasion in prostate cancer cells. *Cancer Res.* **2011**, *71*, 7301–7311.
19. Jinawath, N.; Vasoontara, C.; Jinawath, A.; Fang, X.; Zhao, K.; Yap, K.-L.; Guo, T.; Lee, C.S.; Wang, W.; Balgley, B.M.; et al. Oncoproteomic analysis reveals co-upregulation of RELA and STAT5 in carboplatin resistant ovarian carcinoma. *PLoS. ONE.* **2010**, *5*, e11198, doi:10.1371/journal.pone.0011198.
20. Lee, Y.-J.; Wu, C.-C.; Li, J.-W.; Ou, C.-C.; Hsu, S.-C.; Tseng, H.-H.; Kao, M.-C.; Liu, J.-Y. A rational approach for cancer stem-like cell isolation and characterization using CD44 and prominin-1(CD133) as selection markers. *Oncotarget* **2016**, *7*, 78499–78515, doi:10.18632/oncotarget.12100.
21. Chang, P.-Y.; Liao, Y.-P.; Wang, H.-C.; Chen, Y.-C.; Huang, R.-L.; Wang, Y.-C.; Yuan, C.-C.; Lai, H.-C. An epigenetic signature of adhesion molecules predicts poor prognosis of ovarian cancer patients. *Oncotarget* **2017**, *8*, 53432–53449, doi:10.18632/oncotarget.18515.
22. Park, G.B.; Kim, D. MicroRNA-503-5p inhibits the CD97-mediated JAK2/STAT3 pathway in metastatic or paclitaxel-resistant ovarian cancer cells. *Neoplasia* **2019**, *21*, 206–215, doi:10.1016/j.neo.2018.12.005.
23. Lu, Y.Y.; Sweredoski, M.J.; Huss, D.; Lansford, R.; Hess, S.; Tirrell, D.A. Prometastatic GPCR CD97 is a direct target of tumor suppressor microRNA-126. *ACS Chem. Biol.* **2013**, *9*, 334–338.
24. Mustafa, T.; Eckert, A.; Klonisch, T.; Kehlen, A.; Maurer, P.; Klintschar, M.; Erhuma, M.; Zschoyan, R.; Gimm, O.; Dralle, H.; et al. Expression of the epidermal growth factor seven-transmembrane member CD97 correlates with grading and staging in human oral squamous cell carcinomas. *Cancer Epidemiol. Biomarkers Prev.* **2005**, *14*, 108–119.
25. Chidambaram, A.; Fillmore, H.L.; van Meter, T.E.; Dumur, C.I.; Broaddus, W.C. Novel report of expression and function of CD97 in malignant gliomas: correlation with Wilms tumor 1 expression and glioma cell invasiveness. *J. Neurosurg.* **2012**, *116*, 843–853.
26. Safaee, M.; Clark, A.J.; Oh, M.C.; Ivan, M.E.; Bloch, O.; Kaur, G.; Sun, M.Z.; Kim, J.M.; Oh, T.; Berger, M.S.; et al. Overexpression of CD97 confers an invasive phenotype in glioblastoma cells and is associated with decreased survival of glioblastoma patients. *PLoS. ONE.* **2013**, Apr 26;8(4):e62765. doi: 10.1371/journal.pone.0062765, doi:10.1371/journal.pone.0062765.
27. Liu, J.K.; Lubelski, D.; Schonberg, D.L.; Wu, Q.; Hale, J.S.; Flavahan, W.A.; Mulkearns-Hubert, E.E.; Man, J.; Hjelmeland, A.B.; Yu, J.; et al. Phage display discovery of novel molecular targets in glioblastoma-initiating cells. *Cell Death. Differ.* **2014**, *21*, 1325–1339.
28. Mallawaaratchy, D.M.; Buckland, M.E.; McDonald, K.L.; Li, C.C.; Ly, L.; Sykes, E.K.; Christopherson, R.I.; Kaufman, K.L. Membrane proteome analysis of glioblastoma cell invasion. *J. Neuropathol. Exp. Neurol.* **2015**, *74*, 425–441.
29. Safaee, M.; Fakurnejad, S.; Bloch, O.; Clark, A.J.; Ivan, M.E.; Sun, M.Z.; Oh, T.; Phillips, J.J.; Parsa, A.T. Proportional upregulation of CD97 isoforms in glioblastoma and glioblastoma-derived brain tumor initiating cells. *PLoS. ONE.* **2015**, *10*, e0111532.
30. Eichberg, D.G.; Slepak, T.I.; Pascoini, A.L.; Komotar, R.J.; Ivan, M.E. Genetic manipulation of adhesion GPCR CD97/ADGRE5 modulates invasion in patient-derived glioma stem cells. *J. Neurooncol.* **2021**, *153*, 383–391, doi:10.1007/s11060-021-03778-8.
31. Safaee, M.M.; Wang, E.J.; Jain, S.; Chen, J.-S.; Gill, S.; Zheng, A.C.; Garcia, J.H.; Beniwal, A.S.; Tran, Y.; Nguyen, A.T.; et al. CD97 is associated with mitogenic pathway activation, metabolic reprogramming, and immune microenvironment changes in glioblastoma. *Sci. Rep.* **2022**, *12*, 1464, doi:10.1038/s41598-022-05259-y.
32. Coustan-Smith, E.; Song, G.; Clark, C.; Key, L.; Liu, P.; Mehrpooya, M.; Stow, P.; Su, X.; Shurtleff, S.; Pui, C.H.; et al. New markers for minimal residual disease detection in acute lymphoblastic leukemia. *Blood.* **2011**, *117*, 6267–6276.

33. Mirkowska, P.; Hofmann, A.; Sedek, L.; Slamova, L.; Mejstrikova, E.; Szczepanski, T.; Schmitz, M.; Cario, G.; Stanulla, M.; Schrappe, M.; et al. Leukemia surfaceome analysis reveals new disease-associated features. *Blood* **2013**, *121*, e149-e159.
34. Baraka, A.; Sherief, L.M.; Kamal, N.M.; Shorbagy, S.E. Detection of minimal residual disease in childhood B-acute lymphoblastic leukemia by 4-color flowcytometry. *Int. J. Hematol.* **2017**, *105*, 784–791, doi:10.1007/s12185-017-2206-4.
35. Diamanti, P.; Cox, C.V.; Ede, B.C.; Uger, R.A.; Moppett, J.P.; Blair, A. Targeting pediatric leukemia-propagating cells with anti-CD200 antibody therapy. *Blood Adv.* **2021**, *5*, 3694–3708, doi:10.1182/bloodadvances.2020003534.
36. Saito, Y.; Kitamura, H.; Hijikata, A.; Tomizawa-Murasawa, M.; Tanaka, S.; Takagi, S.; Uchida, N.; Suzuki, N.; Sone, A.; Najima, Y.; et al. Identification of therapeutic targets for quiescent, chemotherapy-resistant human leukemia stem cells. *Sci. Transl. Med.* **2010**, *2*, 17ra9, doi:10.1126/scitranslmed.3000349.
37. Bonardi, F.; Fusetti, F.; Deelen, P.; van, G.D.; Vellenga, E.; Schuringa, J.J. A proteomics and transcriptomics approach to identify leukemic stem cell (LSC) markers. *Mol. Cell Proteomics.* **2013**, *12*, 626–637.
38. Wobus, M.; Bornhauser, M.; Jacobi, A.; Krater, M.; Otto, O.; Ortlepp, C.; Guck, J.; Ehninger, G.; Thiede, C.; Oelschlagel, U. Association of the EGF-TM7 receptor CD97 expression with FLT3-ITD in acute myeloid leukemia. *Oncotarget* **2015**, *6*, 38804–38815.
39. Ho, T.-C.; LaMere, M.; Stevens, B.M.; Ashton, J.M.; Myers, J.R.; O'Dwyer, K.M.; Liesveld, J.L.; Mendler, J.H.; Guzman, M.; Morrissette, J.D.; et al. Evolution of acute myelogenous leukemia stem cell properties after treatment and progression. *Blood* **2016**, *128*, 1671–1678, doi:10.1182/blood-2016-02-695312.
40. Maiga, A.; Lemieux, S.; Pabst, C.; Lavallée, V.-P.; Bouvier, M.; Sauvageau, G.; Hébert, J. Transcriptome analysis of G protein-coupled receptors in distinct genetic subgroups of acute myeloid leukemia: identification of potential disease-specific targets. *Blood Cancer J.* **2016**, *6*, e431, doi:10.1038/bcj.2016.36.
41. Coustan-Smith, E.; Song, G.; Shurtleff, S.; Yeoh, A.E.-J.; Chng, W.J.; Chen, S.P.; Rubnitz, J.E.; Pui, C.-H.; Downing, J.R.; Campana, D. Universal monitoring of minimal residual disease in acute myeloid leukemia. *JCI Insight* **2018**, *3*, doi:10.1172/jci.insight.98561.
42. Vaikari, V.P.; Yang, J.; Wu, S.; Alachkar, H. CD97 expression is associated with poor overall survival in acute myeloid leukemia. *Exp. Hematol.* **2019**, *75*, 64-73.e4, doi:10.1016/j.exphem.2019.06.474.
43. Martin, G.H.; Roy, N.; Chakraborty, S.; Desrichard, A.; Chung, S.S.; Woolthuis, C.M.; Hu, W.; Berezniuk, I.; Garrett-Bakelman, F.E.; Hamann, J.; et al. CD97 is a critical regulator of acute myeloid leukemia stem cell function. *J Exp. Med.* **2019**.
44. Yang, J.; Wu, S.; Alachkar, H. Characterization of upregulated adhesion GPCRs in acute myeloid leukemia. *Transl. Res* **2019**, *212*, 26–35, doi:10.1016/j.trsl.2019.05.004.
45. Chen, C.-T.; Wang, P.-P.; Mo, W.-J.; Zhang, Y.-P.; Zhou, W.; Deng, T.-F.; Zhou, M.; Chen, X.-W.; Wang, S.-Q.; Wang, C.-X. Expression profile analysis of prognostic long non-coding RNA in adult acute myeloid leukemia by weighted gene co-expression network analysis (WGCNA). *J. Cancer* **2019**, *10*, 4707–4718, doi:10.7150/jca.31234.
46. Houtsma, R.; van der Meer, Nisha K; Meijer, K.; Morsink, L.; Hogeling, S.M.; Woolthuis, C.; Ammatuna, E.; Nijk, M.; Boer, B. de; Huls, G.; et al. CombiFlow: Combinatorial AML-specific plasma membrane expression profiles allow longitudinal tracking of clones. *Blood Adv.* **2021**, doi:10.1182/bloodadvances.2021005018.
47. Shen, Y.; Best, O.G.; Mulligan, S.P.; Christopherson, R.I. Ibrutinib and idelalisib block immunophenotypic changes associated with the adhesion and activation of CLL cells in the tumor microenvironment. *Leuk. Lymphoma* **2018**, *59*, 1927–1937, doi:10.1080/10428194.2017.1403598.

48. Gray, J.X.; Haino, M.; Roth, M.J.; Maguire, J.E.; Jensen, P.N.; Yarme, A.; Stetler-Stevenson, M.A.; Siebenlist, U.; Kelly, K. CD97 is a processed, seven-transmembrane, heterodimeric receptor associated with inflammation. *J. Immunol.* **1996**, *157*, 5438–5447.
49. Hamann, J.; Wishaupt, J.O.; van Lier, R.A.; Smeets, T.J.; Breedveld, F.C.; Tak, P.P. Expression of the activation antigen CD97 and its ligand CD55 in rheumatoid synovial tissue. *Arthritis Rheum.* **1999**, *42*, 650–658.
50. Vogl, U.M.; Öhler, L.; Rasic, M.; Frischer, J.M.; Modak, M.; Stöckl, J. Evaluation of prognostic immune signatures in patients with breast, colorectal and pancreatic cancer receiving chemotherapy. *Anticancer Res* **2017**, *37*, 1947–1955, doi:10.21873/anticanres.11535.
51. Wang, C.; Jie, J.; Li, D.; Liu, Y.; Gao, J.; Song, L. Clinical value of CD97 and CD55 levels in the differential diagnosis of tuberculous and malignant pleural effusions. *Medicine* **2021**, *100*, e26771, doi:10.1097/MD.0000000000026771.
52. Hsiao, C.C.; Keysselt, K.; Chen, H.Y.; Sittig, D.; Hamann, J.; Lin, H.H.; Aust, G. The Adhesion GPCR CD97 inhibits apoptosis. *Int. J Biochem. Cell Biol.* **2015**, *65*, 197–208.
53. Xu, X.; Wang, Y.; Mojumdar, K.; Zhou, Z.; Jeong, K.J.; Mangala, L.S.; Yu, S.; Tsang, Y.H.; Rodriguez-Aguayo, C.; Lu, Y.; et al. A-to-I-edited miRNA-379-5p inhibits cancer cell proliferation through CD97-induced apoptosis. *J Clin. Invest* **2019**, *129*, 5343–5356, doi:10.1172/JCI123396.
54. Li, C.; Liu, D.R.; Li, G.G.; Wang, H.H.; Li, X.W.; Zhang, W.; Wu, Y.L.; Chen, L. CD97 promotes gastric cancer cell proliferation and invasion through exosome-mediated MAPK signaling pathway. *World J. Gastroenterol.* **2015**, *21*, 6215–6228.
55. Galle, J.; Sittig, D.; Hanisch, I.; Wobus, M.; Wandel, E.; Loeffler, M.; Aust, G. Individual cell - based models of tumor - environment interactions. Multiple effects of CD97 on tumor invasion. *Am. J. Pathol.* **2006**, *169*, 1802–1811.
56. Wang, T.; Ward, Y.; Tian, L.; Lake, R.; Guedez, L.; Stetler-Stevenson, W.G.; Kelly, K. CD97, an adhesion receptor on inflammatory cells, stimulates angiogenesis through binding integrin counter receptors on endothelial cells. *Blood* **2004**, *105*, 2836–2844.
57. Liu, D.; Trojanowicz, B.; Radestock, Y.; Fu, T.; Hammje, K.; Chen, L.; Hoang-Vu, C. Role of CD97 isoforms in gastric carcinoma. *Int. J. Oncol.* **2010**, *36*, 1401–1408.
58. Ward, Y.; Lake, R.; Faraji, F.; Sperger, J.; Martin, P.; Gilliard, C.; Ku, K.P.; Rodems, T.; Niles, D.; Tillman, H.; et al. Platelets promote metastasis via binding tumor CD97 leading to bidirectional signaling that coordinates transendothelial migration. *Cell Rep.* **2018**, *23*, 808–822, doi:10.1016/j.celrep.2018.03.092.
59. Hilbig, D.; Sittig, D.; Hoffmann, F.; Rothemund, S.; Warmt, E.; Quaas, M.; Sturmer, J.; Seiler, L.; Liebscher, I.; Hoang, N.A.; et al. Mechano-dependent phosphorylation of the PDZ-binding motif of CD97/ADGRE5 modulates cellular detachment. *Cell Rep.* **2018**, *24*, 1986–1995.
